# The frequency of T regulatory cells modulates the survival of multiple myeloma patients: detailed characterisation of immune status in multiple myeloma

**DOI:** 10.1038/bjc.2011.575

**Published:** 2012-01-05

**Authors:** K Giannopoulos, W Kaminska, I Hus, A Dmoszynska

**Affiliations:** 1 Department of Experimental Hematooncology, Medical University of Lublin, 4a Chodzki Street, 20-950 Lublin, Poland; 2 Department of Haematooncology and Bone Marrow Transplantation, Medical University of Lublin, Lublin, Poland

**Keywords:** multiple myeloma, regulatory T cells, dendritic cells

## Abstract

**Background:**

Multiple myeloma (MM) is an immunoproliferative disease characterised by the uncontrolled proliferation of plasma cells, which is accompanied by defects in the immune system.

**Methods:**

This study aimed to characterise the frequency of T regulatory cells (Tregs), dendritic cells (DCs) as well as sub-populations of T cells bearing regulatory properties like CD4^+^GITR^+^, CD4^+^CD62L^+^, CD3^+^TCR*γδ*^+^ along with the concentrations of IL-10, TGF*β*, IL-6 in 66 patients with MM. Subsequently, the influence of therapy on those components of immune system was assessed.

**Results:**

The percentage of both myeloid and plasmacytoid DC was lower in MM compared with control group while Treg (CD4^+^CD25^high^FOXP3^+^) frequencies were significantly higher in MM patients compared with healthy control (6.16% *vs* 0.05%, respectively). Also, the percentages of CD4^+^GITR^+^, CD4^+^CD62L^+^ were increased compared with healthy volunteers. We found that patients with higher percentages of Treg live shorter (median overall survival 21 months *vs* not-reached, *P*=0.013).

**Conclusion:**

This study identifies several abnormalities of immune system in MM, which only partly could be normalised after successful therapy. The dysfunction of immune system such as decreased antigen presentation along with increased frequencies of suppressive cells and cytokines might facilitate progression of the disease and infectious complications limiting survival of MM patients.

Multiple myeloma (MM) is an immunoproliferative disease that is characterised by the uncontrolled proliferation of plasma cells, which is accompanied by defects in the immune system. Initially, abnormalities in number and function of B cells in MM were identified to be responsible for frequent and recurrent infectious complications (Pilarski *et al*, 1984). Although MM is a B-cell malignancy, there are many additional abnormalities in T (Pilarski *et al*, 1989) and NK (Osterborg *et al*, 1990) compartments deepening immunosuppressive status of MM patients. Dendritic cells (DCs) (Banchereau *et al*, 2000) and T regulatory cells (Tregs) (Sakaguchi, 2005) are the most important cells in the immune system, able to control peripheral tolerance as well as response to foreign and tumour antigens. Although myeloid DCs (MDCs) are preferentially involved in initiation and sustainment of immune response, plasmacytoid DC (PDC) maintain peripheral tolerance (Hart, 1997). T regulatory cells are able to control immune responses acting directly and indirectly on antigen presentation as well as effector function of T, B and NK cells (O’Garra and Vieira, 2004; Sakaguchi, 2005; von Boehmer, 2005). Sakaguchi (2005) originally defined Treg as CD4^+^ T cells expressing high levels of IL-2R (CD25). Two sub-populations of Treg might be distinguished, naïve regulatory T cells (nTreg) generated in a thymus as well as induced Treg (iTreg), which on IL-10 and TGF-*β* in periphery acquire regulatory properties (O’Garra and Vieira, 2004; von Boehmer, 2005). The intracellular Forkhead box protein 3 (FOXP3) was identified as a crucial transcription marker that characterises both nTreg and iTreg. Forkhead box protein 3 is required for the suppressive activity and mediates both Treg function and differentiation (O’Garra and Vieira, 2004; von Boehmer, 2005).

This study aimed to characterise the frequency of Treg-expressing FOXP3, sub-populations of DC as well as subsets of T cells bearing regulatory properties like CD4^+^GITR^+^, CD4^+^CD62L^+^, CD3^+^TCR*γδ*^+^ along with the concentration of IL-10, TGF*β*, IL-6 in patients with MM. Subsequently, the influence of therapy and response on those important components of immune system was assessed.

## Materials and methods

### Study subjects and material

The study population consisted of 66 newly diagnosed MM patients (29 women, 37 men, median age 66.5 years; range 39–81) admitted to the Department of Hematooncology and Bone Marrow Transplantation at the Medical University of Lublin. Results obtained in MM were compared with those from 22 healthy volunteers (HVs), with similar sex and age distribution, who served as a control group. Informed consent was obtained from each individual. This study was approved by the local ethics committee. A clinical characteristics of patients is shown in Table 1. Peripheral blood samples were collected at the diagnosis (before therapy), 30 days after third chemotherapy cycle, that is, before fourth chemotherapy cycle and 30 days after sixth cycle. For patients who underwent autologous stem cell transplantation (ASCT), samples were taken before therapy, after third chemotherapy cycle and additional blood sample was taken at day +100 after ASCT.

### Therapy and response assessment

Patients were treated according to Polish Myeloma Study Group Guidelines. Patients eligible for high-dose chemotherapy followed by ASCT (*n*=26) were treated with CTD (cyclophosphamide, thalidomide, dexamethasone) (Dmoszynska *et al*, 2010) regimen while patients ineligible for ASCT received MPT chemotherapy (melphalan, prednisone, thalidomide) (Dmoszynska *et al*, 2009). A total of 49 patients were followed for 3 and 33 days for six cycles of therapy or at day +100 after ASCT. Response was assessed according to International Myeloma Working Group (Durie *et al*, 2006).

### Isolation of the peripheral blood mononuclear cells (PBMCs)

Peripheral blood mononuclear cells were isolated by Biocoll (Biochrom, Berlin, Germany) (density=1.077 g ml^–1^) density gradient centrifugation. Peripheral blood mononuclear cells were washed twice in PBS (Biomed, Lublin, Poland) and then resuspended for future immunostaining. A part of cells was frozen in storage medium (composition: 10% dimetylsulfoxide (AppliChem, Darmstadt, Germany), 20% human albumin (Bioplasma, Bern, Switzerland) and 70% RPMI 1640 medium, (Biochrom AG)) and kept in liquid nitrogen to the time of flow cytometric analysis of Treg.

### Phenotyping of the DCs

To characterise peripheral blood DC sub-populations, immunofluorescence studies were performed using combination of the following monoclonal antibodies (mAbs): anti-human blood DC antigen 1 (BDCA-1) FITC (Miltenyi-Biotec, Bergisch Gladbach, Germany), anti-human BDCA-2 FITC (Miltenyi-Biotec), anti-CD123 PE (Becton Dickinson, New York, NY, USA) and anti-CD19 PE-Cy5 (BD Pharmigen, Hamburg, Germany), anti-CD38 PE (BD Pharmigen) (Dzionek *et al*, 2000). Relevant isotype mouse controls were used (IgG1-FITC, IgG1 PE, IgG1 PE-Cy5, IgG2a-FITC, all form BD Pharmigen). The cells were incubated for 15 min at 4 °C and washed in the PBS afterwards. To minimise FcR-mediated mAbs binding, the cells were stained in the presence of FcR-blocking reagent (Miltenyi-Biotec) containing human IgG. Next, the cells were analysed by flow cytometry. Myeloid DC were characterised as BDCA-1^+^CD19^−^ and PDC were characterised by co-expression of BDCA-2 and CD123. Additionally, CD38 expression was measured on MDC.

### Assessment of Tregs

For the assessment of Treg (CD4^+^CD25^high^FOXP3^+^), PBMC were thawed with RPMI 1640 containing DNA-aze (Sigma-Aldrich, Munich, Germany) with a yield of viable cells >95%. Next, cells were washed twice in PBS containing 2% FCS (Biochrom AG) and counted. After this, 1 × 10^6^ cells were incubated with mAbs for surface staining such as anti-CD25 PE (BD Biosciences, Franklin Lakes, NJ, USA), anti-CD4 PerCP (BD Biosciences), anti-CD8 PerCP (BD Biosciences), anti-CD62L antigen-presenting cell (APC) (BD Biosciences), anti-GITR PE (BioLegend, San Diego, CA, USA) and anti-TCR*γδ* FITC (BD Biosciences). After incubation, the cells were analysed by flow cytometry. We further estimated intracellular FOXP3 expression. Cells were permeabilised, next blocked with rat serum and stained with anti-FOXP3 Alexa Fluor 488 (BioLegend) and relevant isotype rat controls. After the intracellular staining, cells were washed and estimated for the expression of FOXP3 and CD25 on CD4^+^ cells by flow cytometry (100 000 cells were analysed). T regulatory cells were characterised as CD4^+^CD25^high^ expressing FOXP3^+^. Example of analysis of the frequency of Treg of MM patient is displayed in Supplementary Figure 1.

### Cytokine assays

Serum samples were obtained from MM patients in certain time points, that is: before treatment, after three and six cycles of chemotherapy and frozen to the time of analysis. After thawing, serum concentration of IL-6, IL-10 and TGF*β* was measured using commercially available enzyme linked (ELISA) assay kits Quantikine (R&D Systems, Minneapolis, MN, USA) according to the manufacturer's instructions. In this study, we used the Quantikine Human TGF-b1 ELISA Immunoassay (Cat. No. DB100B), which is designed to measure activated TGF-b1 in serum. To activate latent TGF-b1 to the immunoreactive form, we incubated serum with acid solution for 10 min and neutralised the acidified sample according to the manufacturer's protocol.

### Statistical analysis

All results were presented as median values. The Mann–Whitney test was used to evaluate differences between groups of analysed MM patients and HV. The influence of therapy on certain immune parameters was analysed using multiparameter nonparametric Kruskal–Wallis test. Values of *P*<0.05 were considered to be statistically significant. The Kaplan–Meier method and the log-rank test were used to assess overall survival (OS) in different groups of patients.

## Results

### Peripheral blood DCs are decreased in MM patients

**Figure 1:**
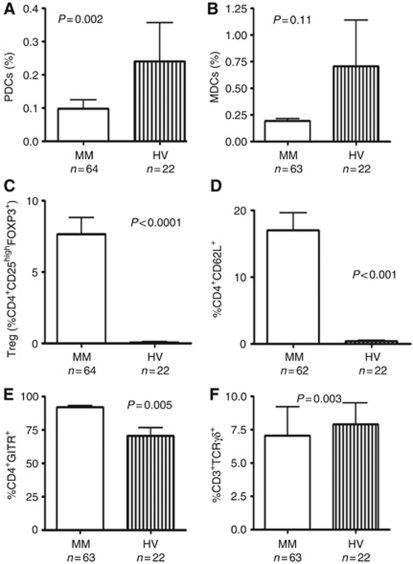
Percentages of PDCs (**A**), MDCs (**B**), Tregs (**C**), CD4^+^CD62L^+^ cells (**D**), CD4^+^GITR^+^ cells (**E**) and CD3^+^TCR*γδ*^+^ (**F**) in MM patients and in HVs. Plasmacytoid DC characterised by coexpression of BDCA-1 and CD123 and MDCs characterised as BDCA-1^+^CD19^−^ were assessed by flow cytometry in fresh isolated PBMCs of MM patients and HVs. Other T-cell subsets possessing regulatory properties were assessed by flow cytometry form frozen PBMCs as described in details in Materials and Methods section.

Plasmacytoid DCs were described as BDCA-2 and CD123 positive. Myeloid DCs were identified as BDCA-1 positive and CD19 negative (BDCA-1^+^/CD19^−^). We observed that the median frequency of PDC was significantly lower in MM patients when compared with HV (0.03% *vs* 0.12% *P*=0.002; Figure 1A). A tendency towards lower frequencies of MDCs was observed in MM patients when compared with HVs (*P*=0.11; Figure 1B). We further estimated the percentage of MDCs characterised by expression of the mobility marker CD38. There was no significant difference between CD38^+^ MDC frequencies in MM patients and HV (79.4% *vs* 69% *P*=0.42). The percentages of PDC and MDC in different patients groups are summarised in Table 2. There was no difference between different patients’ groups characterised by stage, type of monoclonal protein or light chain.

To complete clinical characteristics of DC prevalence in MM patients, we investigated the influence of the frequency of PDC on OS. Patients were divided into two subgroups those with high (above median 0.03%) and low PDC (below or equal median 0.03%) percentages. The Kaplan–Meier analysis revealed no difference in OS between those groups of patients (*P*=0.56). Further patients were analysed according percentages of MDC. We divided MM patients into two subgroups of high and low MDC frequency (above and below median 0.16%, respectively). There was no difference in OS in those groups of MM patients (*P*=0.7339).

### High percentages of Tregs CD4^+^CD25^high^FOXP3^+^ and other sub-populations of lymphocytes bearing regulatory function CD4^+^CD62L^+^, CD4^+^GITR^+^

T regulatory cells were identified as CD4^+^CD25^high^FOXP3^+^. The percentages of Treg among CD4^+^T lymphocytes were significantly higher in MM patients when compared with HV (6.16% *vs* 0.05% *P*<0.0001, Figure 1C). There was no difference between Treg percentages in different patients’ group characterised by stage, type of heavy or light chain. Additionally, we analysed other sub-populations of lymphocytes described to bear regulatory properties like CD4^+^CD62L^+^ and CD4^+^GITR^+^. We found that also these certain population were increased in MM patients. The percentage of CD4^+^CD62L^+^ sub-population reached median 10.35% of CD4^+^ T cells while in healthy control group only 0.425% (*P*<0.001, Figure 1D). The proportion of CD4^+^GITR^+^ among CD4^+^ T lymphocytes in MM patients was upregulated when compared with HV 95.19% *vs* 78%, respectively (*P*=0.005, Figure 1E). There was no correlation of frequencies of these sub-population with age, sex, type of Ig nor with light chain. Neither frequencies of CD4^+^CD62L^+^ and CD4^+^GITR^+^ cells influenced OS of MM patients.

### CD3^+^TCR*γδ*^+^ T-cell sub-population is suppressed in MM patients

The percentage of CD3^+^ lymphocytes expressing *γδ* TCR chains among CD3^+^ T lymphocytes were analysed in the cohort of MM patients. CD3^+^TCR*γδ*^+^ T cells were described both as T cells involved in antigen presentation as well as some researchers defined them as T-cell sub-population of regulatory function. We noted significantly lower percentage of CD3^+^TCR*γδ*^+^ in the CD3^+^ compartment in MM when compared with HV (2.816% *vs* 6.054% *P*=0.003; Figure 1F). There was no difference between different patients’ groups characterised by stage, type of monoclonal protein or light chain.

### The frequency of Tregs modulates the OS of MM patients

**Figure 2:**
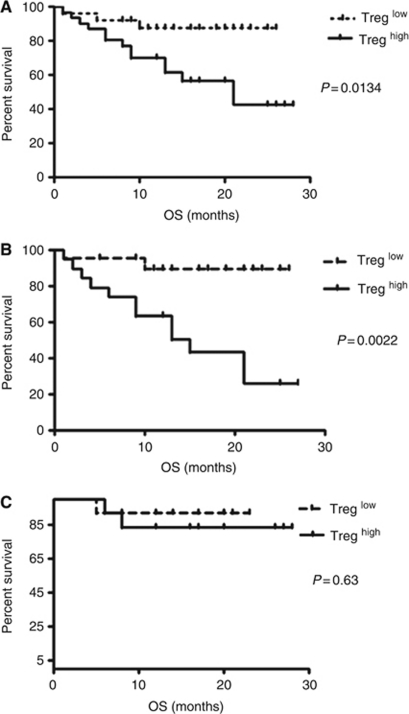
The frequency of Tregs modulates the survival of MM patients. Kaplan–Meier curves displays the OS of groups of MM patients divided according the frequency of Treg. Patients with the percentages of CD4^+^CD25^high^FOXP3^+^ Tregs below median value were classified to Treg low group whereas those of frequencies equal above median value to Treg high group. (**A**) In cumulative analysis, MM patients of Treg high group lived significantly lower as compared with those with lower Treg frequencies. In sub-analysis, the difference in survival was observed only in patients who were treated with MPT (**B**) while in patients treated with CTD followed by ASCT there was no difference in OS in groups of patients characterised by high or low Treg percentages (**C**).

Patients were divided into two cohorts based on the median Treg frequency. Those who have high percentages (equal or above median 6.16%) of Treg lived significantly shorter as compared with those with lower Treg frequencies (below median 6.16%, *P*=0.0134). The median of survival of the low Treg frequency group was 21 months while low Treg frequency group did not reached median value at median follow-up of 32 months (Figure 2A). In sub-analysis, the difference in survival was observed only in patients who were not transplanted (*n*=40, Figure 2B). Similarly, those who have higher percentages of Treg lived significantly shorter as compared with those with lower Treg frequencies (median not reached *vs* 18 months, *P*=0.0134). Interestingly, in patients who undergo ASCT there was no difference in OS in groups of patients characterised by high or low Treg percentages (*n*=26, Figure 2C).

In group of patients who were treated with CTD regimen followed by ASCT, three patients died because of infections (3 out of 3). In group of patient ineligible for ASCT procedure, 14 patients died because of infections (6 out of 14), progression (4 out of 14) and renal failure (4 out of 14). Notably, higher percentages of Treg were observed in patients who died because of infectious complications (7.84% *vs* 5.77% *P*=0.04).

### Evaluation of cytokine serum levels in MM patients

To complete characteristics of immune system in MM patients, we analysed serum levels of cytokines that modulate immune responses. We measured cytokines that were described as able to induce Treg like IL-10, TGF*β*, IL-6 or cytokines that suppress immune responses like IL-10, TGF*β*. We observed significantly higher IL-6 serum levels in MM patients when compared with control group (*P*=0.006; Table 3). Similarly, we noted higher TGF*β* concentration in MM *vs* HV (*P*=0.000001; Table 3). We also observed tendency to higher serum levels of IL-10 (*P*=0.077). Serum concentrations of cytokines were summarised in Table 3. Next, we correlated cytokine serum levels with percentage of Treg. We did not note any correlation between percentage Treg and serum level of IL-10 (*r*^2^=0.29), IL-6 (*r*^2^=0.21) as well as TGF*β* (*r*^2^=0.03).

### Longitudinal analysis of the DCs, Tregs and serum levels of IL-6, IL-10 and TGF*β*

Longitudinal analyses of certain components of immune system were performed on peripheral blood samples collected at the diagnosis (before therapy), 30 days after third chemotherapy and 30 days after sixth cycle. For patients who underwent ASCT, samples were taken before therapy, after third chemotherapy cycle and additional blood sample was taken at day +100 after ASCT. To illustrate the general influence of the therapy on immune system results of latter points of analyses, that is, after six cycles and at day +100 after ASCT were analysed cumulatively.

Before treatment, the median percentage of PDC was 0.09% ±0.17. After three cycles of therapy, we observed decreased median percentage 0.04% ±0.56. We further observed an increase in the median percentage of PDC at the latter point of analyses, that is, after six cycles of therapy or at day +100 after ASCT when compared with the percentage of these cells after three cycles 0.087% *vs* 0.04% *P*=0.03. Comparing percentages before and after six cycles of therapy, we did not observe significant difference. There was no difference in percentages of PDC in responders and non-responders after three or six cycles of therapy nor between patients treated with MPT or ASCT.

**Figure 3:**
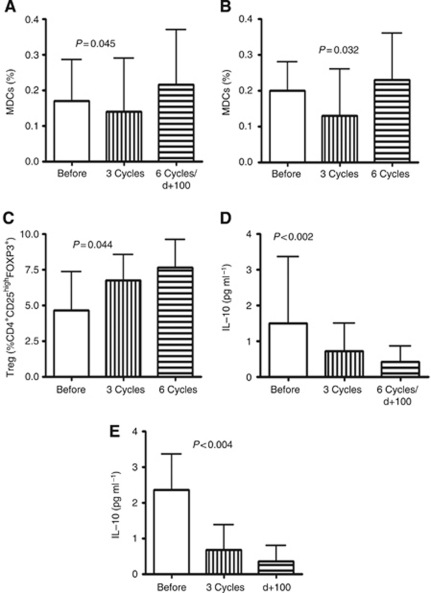
Influence of chemotherapy on certain immune system parameters. Changes in the frequency of the MDCs before, after three cycles (combined MPT for transplant ineligible and CTD for transplant eligible) and at the latter point of analysis, which combined results after six cycles of MPT regimen and day +100 after ASCT (**A**); sub analysis of MDC percentages in the group of patients treated with MPT (**B**); changes in the frequency of the CD4^+^CD25^high^FOXP3^+^ Tregs before, after three cycles and after six cycles of MPT therapy (**C**); changes in the concentrations of the IL-10 before, after three cycles and at the latter point of analysis, which combined results after six cycles in patients treated with MPT regimen and day +100 after ASCT (**D**); and sub-analysis of IL-10 levels in the group of patients who underwent ASCT (**E**).

Significantly higher percentages of MDC at the latter point of analyses, that is, after six cycles of therapy or at day +100 after ASCT when compared with frequency of MDC before treatment (0.21% *vs* 0.16% Figure 3A) were found. Myeloid DCs were decreased after three cycles as compared with frequency of these cells at diagnosis (0.16% *vs* 0.15% Figure 3A). In subgroup analysis, similar changes were observed in patients treated with MPT (Figure 3B) while in group who underwent ASCT there was no change in the frequency of MDC.

Although the Treg frequencies tend to be lower after three cycles of chemotherapy and increased in the latter point of analysis, that is, after six cycles or at day +100 after ASCT (*P*=0.09), observed fluctuations were not significant.

In sub-analyses, while changes of Treg frequencies in patients treated with CTD followed by ASCT were not significant (*P*=0.53), Treg percentages increased in group of patients treated with MPT regimen (*P*=0.044; Figure 3C).

After therapies, the highest values of Tregs (median=13.31%) were observed in patients who progressed and did not responded to treatment (*P*=0.08).

We observed lowering of serum level of IL-6 after three cycles when compared with serum level before treatment (1.68 *vs* 2.31 pg ml^–1^; *P*=0.023). Next, we noted nonsignificant increase serum level of this cytokine at the latter point of analysis to the median value of 2.1 pg ml^–1^. Comparing with serum concentration IL-6 before treatment (median=2.31 pg ml^–1^), we observed tendency to lower IL-6 levels after 6 months of therapy (median=2.10 pg ml^–1^; *P*=0.125). We observed significantly higher concentration of IL-6 after six cycles comparing with serum concentration of this cytokine after three (2.1 *vs* 1.68 pg ml^–1^; *P*=0.014). There was no difference in changes of IL-6 levels in responders and non-responders after three and six cycles of therapy.

We noted statistically significant lowering of IL-10 after three cycles and at the latter point of analysis comparing with serum levels before treatment (1.49 pg ml^–1^ before treatment, 0.72 pg ml^–1^ after three cycles and 0.41 pg ml^–1^ after six cycles of therapy; Figure 3D). In subgroup analysis, similar changes were observed in patients who underwent ASCT (Figure 3E) while in group treated with MPT regimen there was no change in the IL-10 concentrations. There was no difference in changes of IL-10 levels in responders and non-responders after three and six cycles of therapy.

Analysing TGF*β* levels during therapy, we found nonsignificant fluctuation (30 401.8 pg ml^–1^ before therapy, 36 576.7 pg ml^–1^ after three cycles and 25 755.3 pg ml^–1^ at the latter point of analysis). There was no difference in changes of IL-6 levels in responders and non-responders after three and six cycles of therapy nor between patients treated with MPT or ASCT.

## Discussion

In this study, we identified several immune cells abnormalities in patients with MM. On one hand, low frequencies of both MDC and PDC as well as CD3^+^TCR*γδ* T cells reflect impaired antigen presentation. On the other hand, increased frequencies of Tregs and T cells possessing regulatory function might be another highlight of immunosuppression of MM patients that additionally impair immune function. In the first original report on Treg in MM patients, Prabhala *et al* (2006) found increased percentages of Treg in patients with MGUS as well as MM when Treg cells were characterised as by CD25^high^ expression on CD4^+^ T cells. Strikingly, when expression of FOXP3 molecule was analysed, reduced frequencies of CD4^+^FOXP3^+^ T cells in MM patients were observed. Confocal microscopy and western blot analyses further confirmed those results obtained by flow cytometry. In proliferation assays, Treg effectively inhibited proliferation of anti-CD3-stimulated T cells in HVs but not in MM patients pointing to functional impairment of Treg in MM. Study of Prabhala *et al* (2006) provides the unique evidence on reduced frequencies of Treg in patients with haematological malignancies. In contrast, Beyer *et al* (2006) found increased frequencies of Treg when compared with those of healthy controls, both characterised as CD4^+^CD25^high^ and CD4^+^CD25^high^FOXP3^+^. These cells were positive for other Treg markers, such as CTLA-4, CD62L, GITR, OX40, TGF-*β* as well as IL-10. This study could confirm results obtained by Beyer *et al* (2006) in a larger cohort of 66 MM patients. Excess of Treg might result from the influence of inflammatory cytokines in the microenvironment that mature DC, which effectively induce not only cytotoxic T cells but also Tregs (Banerjee *et al*, 2006). Several types of cytokines were identified in myeloma microenvironment including inflammatory molecules with a key role of IL-6 (Klein and Bataille, 1992) as well as angiogenic factors like VEGF (Yang *et al*, 2009) and TGF-*β* (Zhou *et al*, 2008).

We also analysed clinical significance of Treg in MM patients. We found that patients who had lower frequency of Treg live longer when compared with those with high frequencies of Treg. Interestingly, the difference in survival was observed only in patients who were not transplanted. This important finding suggest of certain role of Treg in facilitation of disease progression and infectious complications. Notably, higher percentages of Treg were observed in patients who died because of infectious complications. Reflecting our data to the large cohort study of ECOG by Kay *et al* (2001), we provide evidence that not only the absolute number of CD4^+^ T cells is of prognosis for MM patients but its composition including proportion of the CD4^+^CD25^high^FOXP3^+^ Treg among CD4^+^. Next, we questioned whether with the use of therapy we could normalise the frequencies of Treg. We could not find reduction of Treg after therapy, which is in line with results obtained by Beyer *et al* (2006) who also did not find changes of Treg frequencies after chemotherapy and allogeneic stem cell transplantation (SCT). Interestingly, they found that ASCT procedure could significantly reduce the percentage of Treg. In our study, we could not observe the influence of different therapeutic regimens (MPT or CTD) on the Treg frequencies nor ASCT reduced Treg frequencies significantly.

Functionally, Treg are also potent inhibitors of APCs including DCs. The reduced number of circulating MDC and PDCs characterised by low HLA-DR, CD40 as well as CD80 expression was found in patients with MM (Brimnes *et al*, 2006) and also in this study. Ratta *et al* (2002) found that DCs were functionally defective showing impaired capacity to stimulate allogeneic T-cell proliferation. Investigators attributed this deficit to high levels of IL-6, because IL-6 could inhibit the colony growth of CD34^+^ DC progenitors and switched the commitment of CD34^+^ cells from DC to monocytic cells showing potent phagocytic activity but no antigen-presentation capacity. Noteworthy, the effect of IL-6 was reversible. In this study, we extend characterisation of DC compartments in MM patients. Although we found lower frequencies of both PDC (*P*=0.002) and MDC (*P*=0.11) in MM patients when compared with HV, there was no significant difference in expression of the mobility marker CD38 on MDC (*P*=0.42). Those results suggest that functional impairment of MDC is mainly due to microenvironmental factors.

Earlier studies also demonstrated reduced number of MDC and PDC as well as increased number of Treg in patients with MM (Beyer *et al*, 2006; Brimnes *et al*, 2006). Such defects of the immune system might be responsible for disease progression and might facilitate infection complications during the course of disease. Impaired number and function of APC as well as impairment of effector T cells coexisting with the excess of Treg might designate novel strategies for therapy. Therapy targeting IL-6 might support the defective antigen presentation in MM patients. Moreover, successful manipulation of the immune system might reduce the number of Treg enabling immune-mediated rejection of tumour cells. To test this hypothesis, we evaluated the influence of therapy and response on certain parameters of immune system. We observed no difference between responders and non-responders according to IL-6, IL-10 and TGF-*β* cytokines levels. The therapy normalised MDC frequencies. We further observed higher frequencies of Treg after six cycles of MPT therapy achieving the highest values of those patients who do not respond to therapy and disease progressed. Quach *et al* (2008) reported on decreased numbers of Treg that are normalised after lenalidomide therapy. In contrast, we found in CLL patients treated with fludarabine + thalidomide regimen reduced frequencies of Treg after effective therapy (Giannopoulos *et al*, 2009).

In conclusion, our results identified several abnormalities of immune system in MM. The dysfunction of immune system such as decreased antigen presentation along with increased frequencies of suppressive cells and cytokines might facilitate progression of the disease and infectious complications. The most important finding of our study is the key function of Treg in modulation of OS of MM patients.

## Figures and Tables

**Table 1:** Clinical characteristics of multiple myeloma patients

*Sex*	
Males	37 (56.1%)
Females	29 (43.9%)
	
*Age*	
Median	66.5
Range	39–81
	
*Durie–Salmon stage*	
I	0 (0%)
II	23 (34.8%)
III	43 (65.2%)
	
*ISS score*	
I	4 (6.1%)
II	21 (31.8%)
III	41 (62.1%)
	
*Monoclonal protein*	
IgG	39 (59.1%)
IgA	13 (19.7%)
IgM	1 (1.5%)
LCD	10 (15.2%)
Non-secreting	3 (4.5%)
	
*β*_*2*_ *-Mikroglobulin (mg l* ^*–1*^ *)*	
Median	6.59
Range	2.04–49.16
	
*Haemoglobin (g dl* ^*–1*^ *)*	
Median	9.45
Range	5.1–14.9
	
*Albumin (g dl* ^*–1*^ *)*	
Median	3.15
Range	1.00–4.65

Abbreviations: Ig=immunoglobulin; ISS=International Staging System; LCD=light chain disease.

**Table 2:** Immune cells according to clinical parameters of multiple myeloma patients

			**Immunoglobulin heavy chain type**		**Light chain type**	
**Immune cell sub-population**	**Stage 2 Durie–Salmon**	**Stage 3 Durie–Salmon**	**IgA**	**IgG**	**Kappa**	**Lambda**
*PDC (%)*						
Median	0.02	0.04	0.02	0.15	0.03	0.04
Range	0.01–0.89	0.01–1.23	0.01–0.04	0.02–0.58	0.01–1.23	0.01–0.89
						
*MDC (%)*						
Median	0.15	0.16	0.14	0.15	0.16	0.16
Range	0.04–0.58	0.01–0.69	0.01–0.69	0.02–0.58	0.01–0.69	0.03–0.40
						
*Treg (%)*						
Median	6.29	6.16	5.88	5.83	5.88	6.19
Range	1.88–14.86	0.45–57.84	1.79–10.34	0.45–20.57	0.45–47.88	1.79–14.70

Abbreviations: Ig=immunoglobulin; MDC=myeloid dendritic cell; PDC=plasmacytoid dendritic cell; Treg=T regulatory cell.

**Table 3:** Serum level of cytokines in group of patients with MM and HV

**Cytokine**	**MM**	**HV**
*IL-6 (pg ml* ^*–1*^ *)*		
Median	3.74	2.124
Range	0.48–40.42	0.5525–4.00
		
*IL-10 (pg ml* ^*–1*^ *)*		
Median	1.16	0.91
Range	0.31–31.34	0.04–1.92
		
*TGFβ (pg ml* ^*–1*^ *)*		
Median	32 233.50	3877.23
Range	6418.13–98 215.90	392.079–10 629.3

Abbreviations: HV=healthy volunteer; IL=interleukin; MM=multiple myeloma; TGF=transforming growth factor.
