# Supplementary figures and images for: The frequency of T regulatory cells modulates the survival of multiple myeloma patients: detailed characterisation of immune status in multiple myeloma
Source: Br J Cancer. 2012 Jan 5;106(3):546–52. doi: 10.1038/bjc.2011.575 (PMC3273338; doi:10.1038/bjc.2011.575)

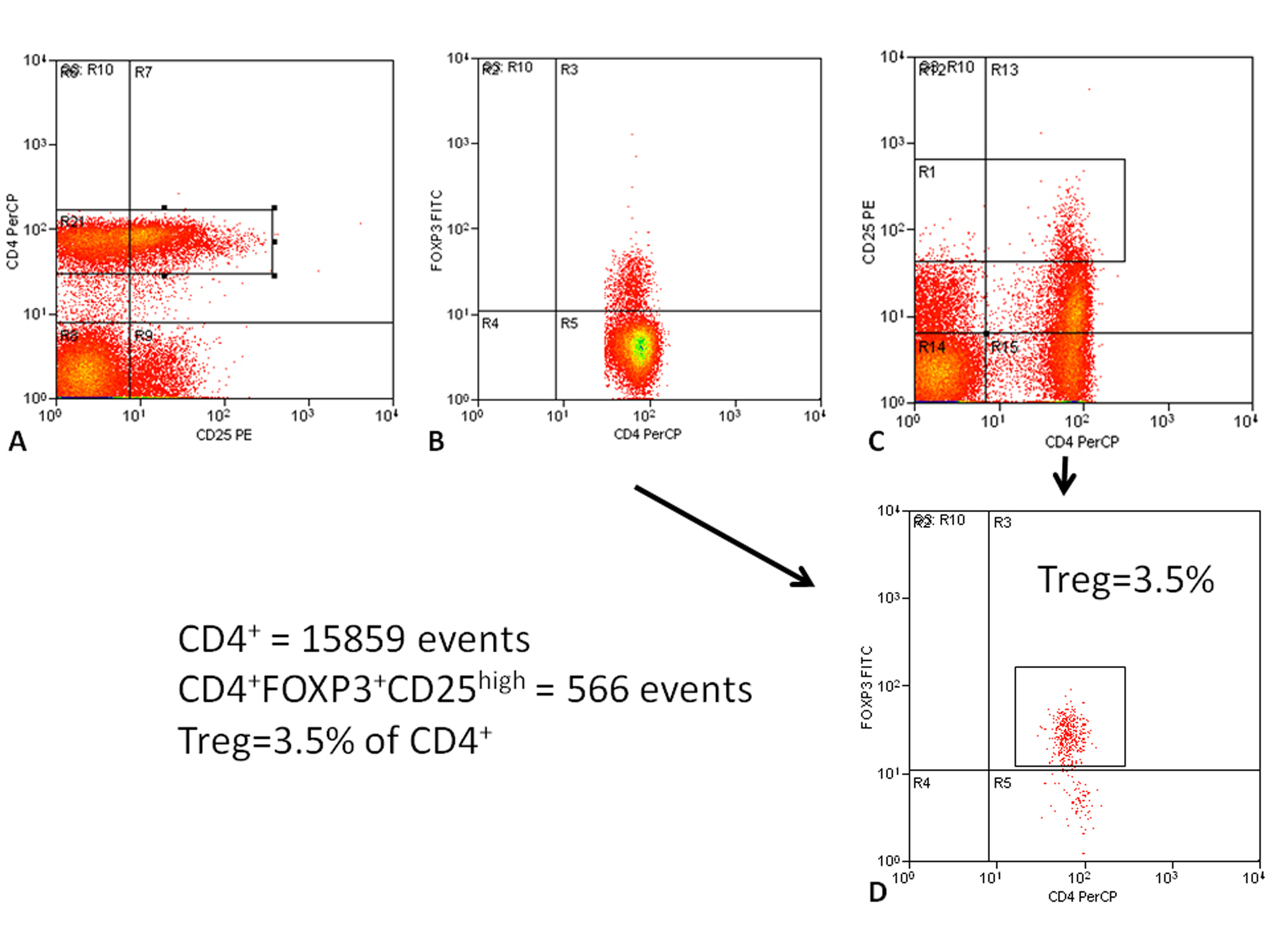

Supplement: Supplementary Figure 1 [file bjc2011575x1.tif]
