# Supplementary material for: The frequency of T regulatory cells modulates the survival of multiple myeloma patients: detailed characterisation of immune status in multiple myeloma
Source: Br J Cancer. 2012 Jan 5;106(3):546–52. doi: 10.1038/bjc.2011.575 (PMC3273338; doi:10.1038/bjc.2011.575)
Supplement: Supplementary Figure Legend [file bjc2011575x2.doc]

Supplementary Figure 1. Example of analysis of the frequency of Treg of MM patient

Example of flow cytometry analysis and calculation of T regulatory cells (Treg) frequency in multiple myeloma patient. Lymphocytes identified on forward scatter (FSC) vs. side scatter (SSC) parameters and gated as CD4^+^ (A). the frequency of T regulator cells was calculated as number of events of cells gated on CD4^+^CD2^5high^FOXP3^+^ among number of CD4^+^ cells. Only cells positive for FOXP3^+^ (B) with high expression of CD25 (C) were analyzed (D).
